# Analysis of complication risk factors in preoperative computed tomography-guided hookwire location of pulmonary nodules

**DOI:** 10.1186/s40001-024-01970-w

**Published:** 2024-07-16

**Authors:** Chuntong Yin, Yu Chen, Renquan Zhang, Anguo Chen, Hanlin Fang, Wenjian Liu, Kai Cui, Zhengqiao Wang, Huaguang Pan

**Affiliations:** Department of Thoracic Surgery, The First Affiliated Hospital of Anhui Medical University, Hefei, 230022 China

**Keywords:** Hookwire, CT guided, Complication, Pulmonary nodules

## Abstract

**Background:**

This study aimed to explore the efficacy of hookwire for computed tomography (CT)-guided pulmonary nodule (PN) localization before video-assisted thoracoscopic surgery (VATS) resection and determine the risk factors for localization-related complications.

**Methods:**

We enrolled 193 patients who underwent preoperative CT-guided PN hookwire localization. The patients were categorized into groups A (103 patients had no complications) and B (90 patients had complications) according to CT and VATS. Uni- and multivariate logistic regression analyses were used to identify risk factors for localization-related complications. A numerical rating scale was used to evaluate hookwire localization-induced pain.

**Results:**

We successfully performed localization in 173 (89.6%) patients. Pneumothorax was the main complication in 82 patients (42.5%). Patient gender, age, body mass index, tumor diameter, consolidation tumor ratio, pathologic diagnosis, position adjustment during location, lesion location, waiting time for surgery, and pleural adhesions were not significantly different between the two groups. The number of nodules, number of punctures, scapular rest position, and depth of insertion within the lung parenchyma were significant factors for successful localization. Multivariate regression analysis further validated the number of nodules, scapular rest position, and depth of insertion within the lung parenchyma as risk factors for hookwire-localization-related complications. Hookwire localization-induced pain is mainly mild or moderate pre- and postoperatively, and some patients still experience pain 7 days postoperatively.

**Conclusions:**

Hookwire preoperative PN localization has a high success rate, but some complications remain. Thus, clinicians should be vigilant and look forward to further improvement.

## Introduction

The detection rate of small pulmonary nodules (PNs), especially ground-glass opacities, has increased significantly with the development of early diagnosis and treatment of lung cancer and the wide application of low-dose computed tomography. Most high-risk PNs can be diagnosed and resected using video-assisted thoracoscopic surgery (VATS). One of the challenges in treating small pulmonary lesions is confirming their location [1]. This causes a transition to thoracotomy or nodule removal by extended resection, resulting in unnecessary trauma to the patient. Accurate PN removal is achieved with the smallest resection range, and healthy lung tissue can be preserved to the greatest extent if the PNs are positioned well preoperatively.

Various techniques for PN localization have been described, including finger palpation, intraoperative ultrasound, and computed tomography (CT)-guided localizer insertion (hook wire, microcoils, methylene blue, Lipiodol, or radionuclides) [1–5]. Each of these methods has advantages and disadvantages. CT-guided hookwire localization technology has become a relatively widely used operation in China because of its vast indications, convenience, low cost, and relatively short operation time. An increasing number of studies have demonstrated the safety of the hookwire localization technology [5–7]. However, complications remain [8, 9]. This study retrospectively analyzed the data of 193 consecutive patients who underwent preoperative CT-guided hookwire localization VATS for PN in our hospital from January 1, 2021, to August 31, 2023, and analyzed the related risk factors for complications.

## Methods

### Patients

The Ethics Committee of the First Affiliated Hospital of Anhui Medical University reviewed and approved this study (Quick-PJ 2023–12–30). Preoperative CT-guided hookwire localization was indicated when thoracic surgeons considered the difficulty in PN detection during VATS. The feasibility of localization and VATS were determined through multidisciplinary discussion, and all patients provided informed consent for PN localization. The clinical practice guidelines, from January 1, 2021, to Aug 31, 2023, reported that 193 consecutive patients underwent VATS with preoperative CT-guided PN localization, which was either ≤ 20 mm in size, ≥ 5 mm in depth, or ground glass nodule [10, 11]. Table 1 shows relevant clinical data of the patients.

**Table 1:** Patient-based characteristics

Parameters	Total(*n* = 193)	Group A(*n* = 103)	Group B(*n* = 90)	*P*-value
Male, *n* (%)	88(45.6%)	44(42.7%)	44(48.9%)	0.393
Age(years)	62.08 ± 10.28	61.81 ± 10.94	62.39 ± 9.52	0.695
BMI(kg/m^2^)	22.04 ± 2.77	22.09 ± 2.92	21.98 ± 2.61	0.790
Tumor diameter(mm)	11.39 ± 3.15	11.35 ± 3.18	11.44 ± 3.14	0.857
Consolidation tumor ratio				0.471
≤ 0.5	142(73.6%)	78(75.7%)	64(71.1%)	
> 0.5	51(26.4%)	25(24.3%)	26(28.9%)	
Number of nodules, *n*(%)				0.013
One	179(92.7%)	100(97.1%)	79(87.8%)	
More than one	14(7.3%)	3(2.9%)	11(12.2%)	
Times of punctures, *n*(%)				0.000
Once	157(81.3%)	100(97.1%)	57(63.3%)	
More than once	36(18.7%)	3(2.9%)	33(36.7%)	
Malignant lesion, *n*(%)	177(91.7%)	94(91.3%)	83(92.2%)	0.811
Scapular rest position, *n*(%)				0.035
Yes	160(82.9%)	91(88.3%)	69(76.7%)	
No	33(17.1%)	12(11.7%)	21(23.3%)	
Position adjustment during location, *n*(%)				0.131
Yes	5(2.6%)	1(0.9%)	4(4.4%)	
No	188(97.4%)	102(99.1%)	86(95.6%)	
Lesion location, n(%)				0.797
Right lung	129(66.8%)	68(66.0%)	61(67.8%)	
Left lung	64(33.2%)	35(34.0%)	29(32.2%)	
Insertion depth within the lung parenchyma, *n*(%)				0.009
< 1cm	29(15.0%)	9(8.7%)	20(22.2%)	
≥ 1cm	164(85.0%)	94(91.3%)	70(77.8%)	
Waiting time for operation(min)	109.21 ± 37.09	106.12 ± 36.08	112.76 ± 38.12	0.216
Pleural adhesions, n(%)	20(10.4%)	9(8.7%)	11(12.2%)	0.431

### Preoperative localization procedures

**Fig. 1:**
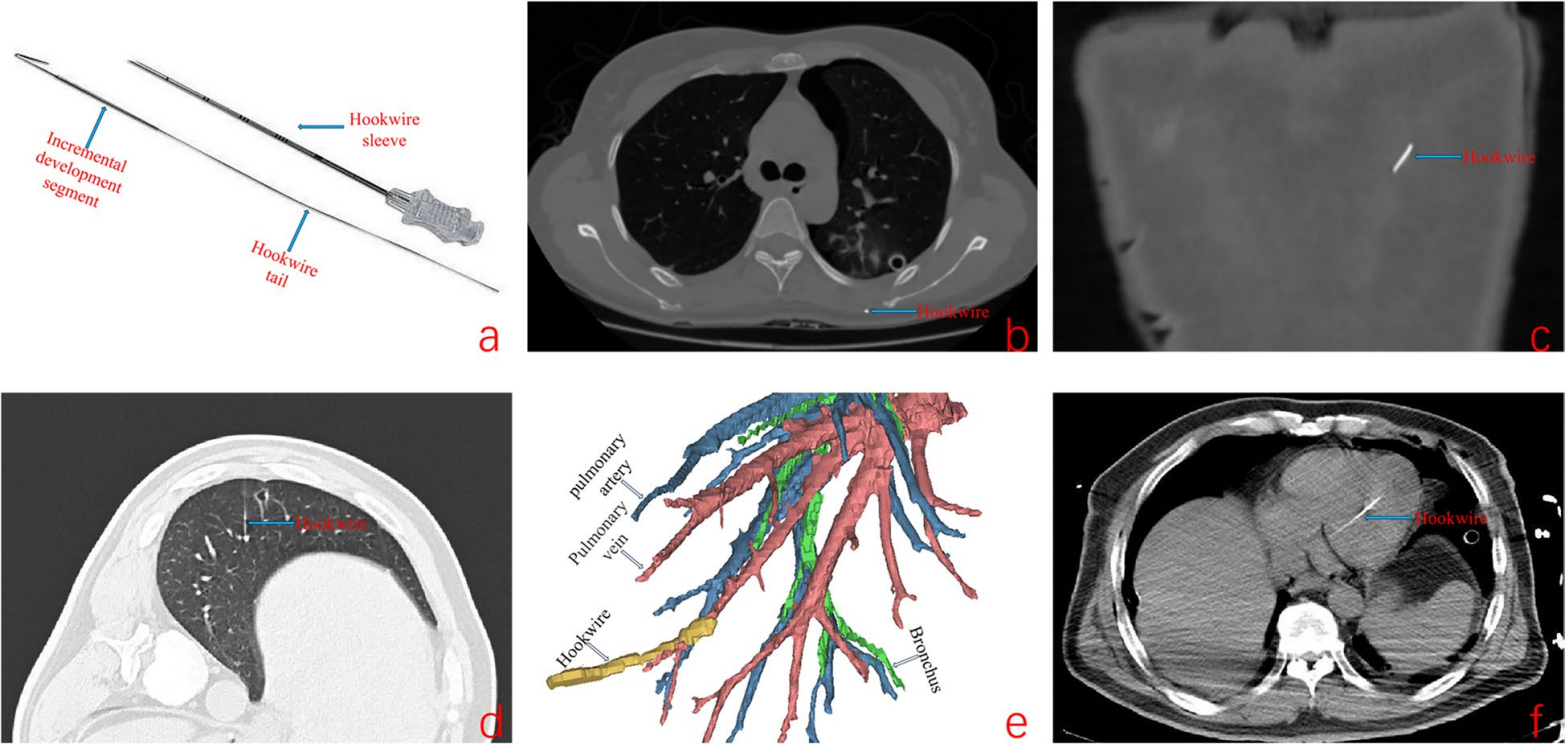
Preoperative CT-guided hookwire location of pulmonary nodules. **a** Schematic of the hookwire (20 G × 120 mm, PAJUNK^®^, Geisingen, Germany); **b**, **c** residual hookwire were found in the transverse and coronal plane of CT; **d**, **e** CT scan after localizer placement and CT scan three-dimensional reconstruction; f emergent CT scan during operation

Experienced thoracic surgeons and radiologists performed all localization procedures together. Patients were placed in supine, lateral, or prone positions according to the location of the nodules on previous CT images. Upper limb encircling, backward extension, or other positions were used, if necessary, to fully expose the nodules. The optimal entry point, angle, and depth of the access route were determined by CT scan while avoiding large vessels, bone structure, emphysema area, or bronchus. The needle was advanced as planned after sterilization and local anesthesia (20 G × 120 mm, PAJUNK^®^, Geisingen, Germany, Fig. 1a). Additional CT scans were performed to ensure that the needle tip was within a radius of 6–10 mm around the nodule, and the angle and depth were adjusted when appropriate [2]. The needle was then carefully withdrawn. A portion of the hookwire outside the skin was cut off and covered loosely with cotton gauze. After localization, the patient was admitted directly to the operating room or given nasal tube oxygen in the ward. And then the electrocardiogram (ECG), oxygen saturation will be monitored, if necessary, drainage tubes also placed.

### Surgical procedure

The patients were anesthetized with single-lung ventilation in the lateral decubitus position. An incision of 3 cm in length was usually made in the 4th–5th intercostal space at the midaxillary line while avoiding the puncture point of the hookwire. An observation port may be added in the 7th–8th intercostal space at the posterior axillary line in patients with dense pleural adhesions. Wedge resection or segmentectomy with a margin of < 2 cm was initially performed for each nodule. Additional resection, such as lobectomy and/or mediastinal lymph node dissection, was considered if infiltrating pulmonary carcinoma was revealed in the frozen specimen. Other present and malignant PNs, which were not localized preoperatively (considered as palpable nodules), were also resected. All specimens will be subjected to a final postoperative pathological diagnosis.

### Pain evaluation

The numerical rating scale (NRS) was used to evaluate pre- and postoperative pain. The pain intensity was classified as no pain (NRS = 0), mild (NRS = 1–3), moderate (NRS = 4–6), or severe pain (NRS = 7–10). Patients were instructed to report their pain scores during calm breathing.

### Statistics

All statistical analyses were performed using Statistical Package for the Social Sciences version 22.0 (IBM, Armonk, New York) software. Continuous variables were expressed as mean ± standard deviation. Student’s t and Pearson *χ*^2^ tests were used to analyze continuous and categorical variables between the groups, respectively. Logistic regression analysis was used for multivariate analysis. A P-value of < 0.05 indicated statistical significance.

## Results

This study revealed that 193 patients (88 males/105 females, mean age: 62.08 ± 10.28 years, mean body mass index [BMI]: 22.04 ± 2.77 kg/m2) underwent resections of preoperatively localized PN by CT-guided hookwire from January 1, 2021, to August 31, 2023 (Table 1). Hookwire-related complications have been indicated to include pneumothorax, bleeding, hemoptysis, displacement, air embolism, hookwire residue, etc. (except pain). Of the 193 patients, 103 demonstrated no complications (Group A), and 90 showed complications (Group B).

Among the nodule and demographic factors, patient gender, age, BMI, tumor diameter, consolidation tumor ratio, pathologic diagnosis, position adjustment during location, lesion location, waiting time for operation, and pleural adhesions were not associated with the occurrence of complications on univariate analysis. The number of nodules, punctures, scapular rest position, and depth of insertion within the lung parenchyma were significant factors for successful localization (Table 2). The evaluation of the significant factors on multivariate analysis revealed the number of nodules (*P* = 0.022), scapular rest position (P = 0.014), and insertion depth within the lung parenchyma (*P* = 0.011) as independent factors for successful localization (Table 3).

**Table 2:** Complications related to hookwire placement

Type of complication	Number of patients
Pneumothorax	82(42.5%)
Bleeding or hemoptysis	19(9.8%)
Hookwire detachment or displacement	20(10.4%)
Other complications	1(0.9%)

**Table 3:** Multivariate analysis results

Variable	B	S.E	95% CI	*P* value
Number of nodules	1.569	0.684	1.257–18.326	0.022
Scapular rest position	1.002	0.407	1.227–6.052	0.014
Insertion depth within the lung parenchyma	1.129	0.446	1.291–7.405	0.011

B, beta; S.E, standard error; CI, confidence interval

Table 2 shows the complications associated to hookwire placement. Successful placement of wires and stable localization were identified on VATS exploration in 173 patients (technical success rate: 89.6%). Pneumothorax was the main complication in 82 patients (42.5%). Of the 20 patients (hookwire detachment or displacement, e.g., Fig. 1b–f) with failed localization, most could be assisted by puncture bleeding on the lung surface to PN resections. However, a more serious complication occurred in one patient whose hookwire entered the left ventricle through the pulmonary vein (Fig. 1d–f). We invited a cardiovascular surgeon to remove the hookwire during thoracotomy under cardiopulmonary bypass, and the patient was transferred to the intensive care unit (ICU) for supportive treatment after surgery. Other complications occurred in one patient (Table 2) because the positioning needle was burned into two segments by the electrocoagulation hook during dense pleural adhesion separation.

Pain remains an issue, although this study did not analyze it as a complication. Most patients were discharged two days postoperatively; thus, the NRS was used to evaluate pre- and postoperative pain. Notably, hookwire localization-induced pain is mainly mild or moderate pre- and postoperatively and pain persists in some patients 7 days postoperatively (Table 4).

**Table 4:** Hookwire localization-induced pain evaluation

Variables, *n*(%)	NRS = 0	NRS = 1–3	NRS = 4–6	NRS = 7–10
Preoperative	0(0.0%)	41(21.2%)	136(70.5%)	16(8.3%)
POD1	0(0.0%)	148(76.7%)	44(22.8%)	1(0.5%)
POD2	0(0.0%)	139(72.0%)	53(27.5%)	1(0.5%)
POD7	109(56.5%)	71(36.8%)	13(6.7%)	0(0.0%)

NRS, Numerical rating scale; POD, postoperative day

## Discussion

Finding a safe, effective, economical, and convenient method to locate PN preoperatively has always been a problem for thoracic surgeons. Proper preoperative localization and rapid nodule detection after resection are the keys to shortening the operative time and reducing the scope of lung resection. Failure of localization causes intentional extension of the resection, lobectomy, lesion omission, and thoracotomy. The literature reports that common localization methods have their advantages and disadvantages. Methylene blue diffuses easily and may affect the pathological results of intraoperative freezing [12, 13]. Medical glue causes some patients to cough violently and cough with a peculiar smell due to irritation; in severe cases, it is injected into blood vessels to cause embolism. The large volume of medical glue after solidification expands surgical resection. Radionuclides and fluorescent particles have a high success rate in localization; however, the equipment requirements are high, the price is expensive, and the popularization range is limited [13]. Placing the spring coils requires X-ray positioning during operation, which brings inevitable radiation, and complications such as pneumothorax and hemothorax occur. Intraoperative ultrasound positioning requires complete lung collapse; otherwise, residual gas in the lung will affect the ultrasound results, and the solid composition of the nodules and the operator’s technology will affect the results [14]. The nodule position in the lower lung is prone to large deviations according to the body surface and lung anatomical marker positioning. Electromagnetic navigation bronchoscopy multi-spot dye-marking technique has high accuracy, but it is expensive, its current popularity is poor, and it cannot be widely promoted [15–17]. The three-dimensional reconstruction-guided PN localization technique has great advantages for the segmental resection of PN located in the middle third of the lung, but it will cause greater trauma and complications in the peripheral third of the PN.

CT-guided percutaneous hookwire localization appeared earlier and has been widely used globally. Reportedly, hookwire localization has a high success rate [18]. It was also the most prominent preoperative PN positioning method used in our unit. However, complications caused by this localization technology cannot be ignored. This study summarizes and analyzes the risk factors for related complications to improve the success rate of localization and to reduce the incidences of complications.

The combination of univariate and multivariate analyses revealed that the number of nodules, scapular rest position, and depth of lung parenchymal insertion were independent factors for successful localization. We then conducted a preliminary exploration of the related complications. (1) Pneumothorax: this is the most prominent complication, mostly due to poor hookwire localization, multiple punctures, etc., which damage the visceral pleura. Pneumothorax occurred in up to 42.5% of patients, fortunately, the majority of patients have less than 10% pulmonary compression, and we have not yet encountered patients with severe pneumothorax requiring drainage or further treatment. The presence of numerous pneumothorax may cause inconsistencies between blood gas analysis results and preoperative lung function, which should be relayed to the anesthesiologist in advance to prevent it from being considered a contraindication for surgery; (2) Bleeding or hemoptysis: the prevalent cause, including the rupture of intercostal or pulmonary blood vessels, and hemoptysis, can often resolve by itself. Once hemothorax occurs, hemostasis can be implemented during VATS and does not need to be treated preoperatively; (3) Hookwire detachment or displacement: needle displacement is a serious complication of hookwire positioning and may cause localization failure, although sometimes it can be assisted by puncture bleeding on the lung surface. At this point, the greatest difficulty may be finding the hookwire rather than the surgery itself, which may be in the chest cavity, between interlobular fissures, inside the chest wall, or anywhere else. However, serious complications should be avoided. We encountered two special cases, one with partial hookwire retention in the muscular layer, inner scapula, and was removed under ultrasound guidance (incremental development segment, Fig. 1a–c), and in another patient, with the hookwire traveling along the pulmonary vein to the left ventricle and was removed under cardiopulmonary bypass (Fig. 1d–f). Shi et al. also reported penetrating cardiac injury caused by hookwire [19]; and (4) Other complications such as air embolism have been reported in some cases of hookwire localization, with an incidence of 0.07–0.15% [20]. Our study reported no such complications, which may be associated with the patient’s cough, deep breathing, or positive pressure ventilation on an anesthetic ventilator. Systemic air embolism occurs due to the entry of gas into the systemic circulation from the pulmonary vein through the left atrium and ventricle. The hookwire is burned into two segments by the electrocoagulation hook during the separation of adhesions due to dense pleural adhesions, which appeared in our center. Part of the hookwire remained in the pulmonary vein because it was not removed in advance. The analysis excluded pain due to operational inevitability (approximately 80% of patients complained of pain). However, clinically, patients often complain of severe pain at hookwire localization, which lasts for a week or even longer in some patients. Chronic postsurgical pain (CPSP) can increase postoperative complications, long-term disability, reduced quality of life, and increased healthcare spending [21, 22]. Kong et al. revealed that the preoperative pain stress of hookwire localization increased the incidence and intensity of CPSP rather than acute pain at three months postoperatively, especially in patients with multiple hookwires [23].

In our opinion, several lessons can be learned from these cases to avoid complications in the future. First, the pulmonary vasculature should be avoided as far as possible during the localization procedure. Second, we recommend avoiding vertical needle insertion in the lateral decubitus position as much as possible, as the positioning needle is parallel to the pulmonary vein, which may cause it to enter the blood vessel and displace, although postural position and the occurrence of complications in our data demonstrated no statistically significant difference (*P* = 0.131). Third, the nodule is located at the edge of the lobe fissure and should be localized near the central side of the lobe, where the nodule is located, to prevent the puncture needle from freeing in the oblique fissure and not passing through the lung tissue. Fourth, trying to avoid adjusting the hookwire multiple times, 2 cm away from the nodule, seems acceptable. Fifth, we ensured that the hookwire was inserted at least 1 cm into the lung, especially in the case of pneumothorax, after multiple adjustments. Sixth, after successful positioning, the tail filament outside the skin should be kept as much as possible or not cut off, and should be directly relaxed and fixed on the skin surface. Seventh, transfer to the operating room as soon as possible after completing the positioning. Concurrently, scapula movement is minimized on the location side. Eighth, the hookwire should be pulled out of the lung parenchyma at the beginning of the operation, and the anchor point should be marked with sutures or Hem-o-lok.

Fan et al. developed an innovative improvement to the hookwire. It makes it easier for lung tissue to be firmly positioned, and patients do not need to maintain a specific posture during the waiting time for surgery. Additionally, the absorbable soft suture was well tolerated by patients because no infection, dyspnea, cough, or chest pain were observed after localization, and the patients reported no notable discomfort or pain [4]. However, PN is close to the heart or large blood vessels, the puncture path has a scapula occlusion, and the location near the diaphragm cannot be completed. More improved technology is being developed. Therefore, clinicians should improve their understanding of these complications and remain vigilant.

This study had certain limitations. Some later complications, such as pneumothorax, may not have been recorded because CT was performed only at the time of localization. At the same time, the sample size of this study was little smaller, multicenter clinical studies are still needed.

## Conclusions

Hookwire preoperative PN localization has a high success rate, however, some complications remain, most of which do not require further treatment or can be managed simultaneously during the surgical procedure. We validated the number of nodules, scapular rest position, and depth of insertion within the lung parenchyma as risk factors for hookwire-localization-related complications. Hookwire localization-induced pain is mainly mild or moderate pre- and postoperatively, and pain persists in some patients 7 days postoperatively. Clinicians should be vigilant and look forward to further improvement.

## Data Availability

The datasets used and/or analyzed during the current study are available from the corresponding author on reasonable request.
